# Community care coordination for stroke survivors: results of a complex intervention study

**DOI:** 10.1186/s12913-020-05993-x

**Published:** 2020-12-19

**Authors:** Johannes Deutschbein, Ulrike Grittner, Alice Schneider, Liane Schenk

**Affiliations:** 1 Charité – Universitätsmedizin Berlin, corporate member of Freie Universität Berlin, Humboldt-Universität zu Berlin, and Berlin Institute of Health, Charitéplatz 1, 10117 Berlin, Germany; 2 Institute of Biometry and Clinical Epidemiology, Charité – Universitätsmedizin Berlin, Corporate Member of Freie Universität Berlin, Humboldt-Universität zu Berlin, and Berlin Institute of Health, Charitéplatz 1, 10117 Berlin, Germany; 3 Berlin Institute of Health (BIH), Anna-Louisa-Karsch-Str. 2, 10178 Berlin, Germany

**Keywords:** Care coordination, Stroke, Health care utilization, Readmissions, Costs, Germany

## Abstract

**Background:**

Outpatient follow-up care for stroke survivors is often inadequate and mostly self-organized by the patients themselves. In the German health care system, there are no standard care programs for patients after they are discharged from the hospital to support them with their multifaceted and heterogeneous health care needs. The objective of this complex intervention study was to evaluate the effectiveness of a post-stroke care coordination program in comparison to standard care in the first year after a stroke.

**Methods:**

Patients aged 55 and older who had survived a stroke or a transient ischemic attack (TIA) within the last 6 months before enrollment were included. Participants received care coordination either by telephone or face-to-face for up to 1 year. Patients’ health insurance claims data were used to measure outcomes. The control group consisted of stroke survivors receiving standard care and was constructed by exact matching based on six criteria. Outcome measures were health services utilization, rate of recurrent events, readmissions and accompanying costs, and mortality. Outcomes were tested using different multiple models.

**Results:**

In total, *N* = 361 patients were included in the analyses. Intervention participants had seen an outpatient neurologist more often (OR = 4.75; 95% CI: 2.71–8.31) and were readmitted to a hospital less frequently (IRR = 0.42; 95% CI: 0.29–0.61), resulting in lower hospital costs (IQR = €0–1910 in the intervention group, IQR = €0–4375 in the control group). There were no substantial group differences in the rate of recurrent events and mortality.

**Conclusion:**

This study showed the beneficial potential of care coordination for a vulnerable patient population: the utilization rate of important health services was increased, and the rate of hospital readmissions decreased as a result. Future research should focus on the risk of recurrent strokes and the long-term effects of improved care.

**Trial registration:**

DRKS00017526 on DRKS – German Clinical Trials Register (retrospectively registered: 21 June 2019).

## Background

Stroke remains a leading cause of mortality and the third most common cause of disability worldwide [1]. Due to improved acute care and prevention strategies, mortality and age-adjusted incidence have been decreasing in high-income countries over the last decades. At the same time, the absolute numbers of stroke events in aging populations are still increasing. For the EU region, an increase of 36% between 2000 (1.1 million) and 2025 (1.5 million) was projected in 2006 [2].

Stroke survivors often suffer from a wide range of functional impairments and deficits and usually depend on individualized treatment for many months after the event. Thus, optimal care requires a multidisciplinary approach, including physiotherapy, occupational therapy, speech therapy, and nursing care [3]. In addition, patients are at a high risk of having recurrent strokes and need regular screening and risk factor treatment [4].

However, in many high-income countries such as the US [5], Canada [6], and parts of Europe [7, 8], health care structures for long-term care after stroke are not sufficiently developed and do not fully meet patients’ complex and heterogeneous needs. After being discharged from the hospital, stroke patients and their caregivers are often left alone to manage their health services and navigate the health care systems [9]. This ‘treatment burden’ can be overwhelming for these usually older and vulnerable patients [10].

In Germany, the health care system is characterized by its comprehensive health service supply and universal health coverage. Statutory health insurance companies provide mandatory insurance for about 90% of the German population (the remaining 10% are privately insured) and cover most evidence-based treatments [11]. Yet, there is a firm division between hospital and outpatient care, which impedes an effective communication among providers and leads to the fragmentation of services [11]. Further, outpatient health care providers are unevenly distributed within cities and across regions [12]. There are, for instance, significant disparities in the distribution of primary care physicians, mainly specialists, between urban and rural regions, but also between wealthier and deprived areas within big cities [12]. This poses additional burdens to receive adequate care, particularly for vulnerable patients with limited mobility, such as stroke survivors. Stroke survivors are often unaware of important and beneficial health services available to them, lack knowledge on how to access them, or do not receive treatment in a timely manner. Continuity of care after discharge is not ensured [13–15], and evidence-based recommendations for follow-up care and secondary prevention are not always met [8]. Patients themselves perceive their health care and support as insufficient [16, 17].

To overcome these problems, various strategies and concepts have been discussed by health care providers, politicians, and scholars, such as early supported discharge [18]; patient education programs [19]; behavioral interventions [20]; tele-rehabilitation [21]; and transitional care [22], i.e., the coordination of care during transfers between different healthcare settings. Promising, innovative approaches include care coordination through case management for stroke survivors in the community. Here, additional nurses or social workers support patients individually during a certain period and take responsibility for the coordination of and access to all required treatments and services [23, 24]. Improved interprofessional collaboration between providers, which includes aspects such as direct communication to overcome differences in professional cultures and languages, further joint training, structured referrals of patients, systematic exchange of information, and agreement on care strategies and standards, is regarded as a prerequisite of high-quality health care [25]. However, evidence for the effectiveness of community-based models of post-stroke support is still insufficient. More research is needed, especially in the context of the German health care system.

Against this background, a complex intervention providing systematic care coordination for stroke survivors in the community was developed and implemented. Care coordination was individually provided to patients by the case managers (nurses) and supported by the systematic interprofessional collaboration of health care providers in the district, i.e., the network members.

The aim of this study was to evaluate the intervention’s effects compared to stroke survivors receiving standard care. In detail, standard care patients had access to the same health care resources as the intervention group in principle but did not have the coordinating support provided by this intervention. Using claims data of intervention participants and standard care patients, analyses focused on health services utilization, recurrent events, readmissions and their costs, as well as mortality rates.

## Methods

### Study design

The study used a pragmatic, non-randomized, controlled trial design, wherein participants in two intervention arms were compared with a quasi-control group. This complex intervention consisted of several components combined together to reach a specific aim: improved, patient-centered post-stroke care.

### Study setting

The intervention was implemented in a district of Berlin, Germany, with almost 400,000 inhabitants and set within the structure of a regional health care network. While the network approach is promoted widely in German health policy, there is considerable variety in type, structure, and goals among existing networks. The Berlin network was established in the year 2000 and might be exceptional regarding its bottom-up approach and the variety of involved community providers, ranging from nursing care, physical, occupational, and speech therapy practices, to hospitals. The primary aims of the network were to support the elderly to maintain their independence, further improve the quality of health services, and to strengthen communication between health care providers in the neighborhood. At the time of the stroke project launch in 2013, the network had around 60 members. For this stroke-specific project, community physicians, i.e., general practitioners and neurologists, were asked to join the network and participate in the intervention.

### Participants

Eligible participants included inhabitants of the district who had had a stroke (first or recurrent) or a transient ischemic attack (TIA) within the 6 months prior to enrollment in the study. As the focus of the network was on care for the elderly, participants had to be at least 55 years old and needed to be insured with a statutory health insurance company. Exclusion criteria included being enrolled with a private health insurance company and insufficient German language skills.

Patients were consecutively recruited by all network members, i.e., health services providers of various disciplines and sectors in the district. Most participants were recruited by hospitals and rehabilitation clinics (55%) and outpatient neurologists (16%). In a first step, patients gave written consent allowing the program team to contact them. Subsequently, members of the project team obtained informed consent from each participant or – in case of a cognitive impairment – the legal guardian. This consent included participation in the coordination program and the permission to request and analyze their personal health care records from their health insurance provider. Patient recruitment took 18 months, beginning in January 2014. Participants joined the program for up to 12 months, depending on their needs and preferences.

### Intervention

The intervention addressed the ‘system level’ and the ‘individual level’ [17] of care coordination.

Improved interprofessional cooperation at the broader system level was targeted through regular network conferences, agreement on care standards, and the use of standardized transition forms. The main purpose of the intervention was to establish an ‘individual level’ case management program to coordinate individual patients’ continued care.

Case management was conducted by two part-time nurses with postgraduate degrees in case management. In contrast to different case management models, case managers were employed by the network and, thus, work independently of hospitals or insurance companies. Their role can be described as a consultant or patient advocate regarding adequate post-stroke care and secondary prevention. After thorough patient information and enrollment, case managers assessed the potential level of required support of each participant. Criteria were limitations in the activities of daily living through motor impairments or cognitive deficits; demand of a wide range for health services; severe comorbidities and increased risk of secondary events; lack of social support; and inadequate housing situation, such as insecure and non-accessible homes. Patients were divided into two groups. Those with high screening scores (≥ 2 dimensions) were assigned to one project arm with intensive face-to-face case management (face-to-face group). Less impaired participants were assigned to a distance case management group (telephone group). Both groups were provided with detailed information brochures on strokes, secondary prevention, and available health care providers in their neighborhood where project participants were guaranteed to receive timely appointments. Additionally, the case managers offered educational sessions for both patients and caregivers. The telephone group received individual consultations about health care such as physiotherapy, secondary prevention through neurologists, and potential resources. Participants in this group were contacted for a follow-up every 3 months. Patients in the face-to-face group were visited at home, where a comprehensive individual treatment plan was developed. This plan included the kind of treatments a patient should receive (e.g., physical or occupational therapy), which physicians the patient should see, what kind of support in activities of daily living the patient may need, and for which benefits and assistive equipment they should apply to receive. The participant’s needs were monitored periodically, and, when necessary, the plan was adjusted. Home visits were repeated every 3 months. Moreover, case managers contacted the patients by telephone each month. In between check-ins, case managers were also available via telephone to participants in both groups.

Case management focused on medical counseling and organization of access to required health services. Furthermore, caseworkers supported participants with their applications for (financial) benefits from health insurance companies or social programs.

Irrespective of patients’ enrollment into the project and recommendations of specific health care providers, all participants were free to visit any physicians, therapists, or facilities outside of the network.

### Data

The effectiveness of the intervention was evaluated using the participants’ claims data from their health insurance company. Statutory health insurance companies in Germany keep detailed records of all health services and costs they cover for their clients. This includes sociodemographic data as well as data on service dates and diagnoses. People can choose from a large number of statutory insurance companies that keep their own data management systems. Since the specific membership distribution of our study population was not known in advance and no patient should be excluded for formal reasons such as insurance membership, the aim was to analyze a large subsample of at least two-thirds of all participants. Eventually, we agreed upon terms of cooperation about access to participants’ claims data with four insurance companies.

Data were received in the form of numerous accounting tables. Afterward, data were controlled for consistency and plausibility, synchronized, and merged as far as necessary for analysis.

### Control group

The control group was compiled of compatible stroke patients receiving standard care. This matching approach drew on claims data from all Berlin stroke patients in the years 2012–2015 who were insured with one of the four cooperating insurance companies (*N* = 10,431). The control group was formed using exact matching based on six criteria: sex, age in 5-year classes, stroke type (ischemic or hemorrhagic stroke, TIA, or unclassified), the severity of the stroke, place of residence in a structurally comparable district of eastern Berlin, and state of long-term care dependency before the stroke.

The severity of stroke was described by the proxy variable ‘length of stay in the stroke unit or another hospital ward, since claims data lack clinical information and no alternative standards to model this kind of data have been established so far [26]. In detail, a median split of the length of stay (8 days) was used. Dependency before the stroke was measured by the degree of nursing care dependency that can be applied for by functionally impaired patients to receive benefits and support [27]. Control cases had to match in all six criteria with the respective case from the intervention group. When more cases than needed fit those requirements, control cases were chosen randomly.

To increase the statistical power, the control group was constructed in a ratio of 3:1 compared to the intervention group. As a result, up to three exact twins from the standard care population matched using the six characteristics mentioned above were included in the control group for each program participant.

### Outcome measures

The analysis of effectiveness of the program concentrated on five outcomes, as represented in the patients’ claims records.

1. Utilization of community neurologist services
2. Rate of recurrent strokes
3. Number of all readmissions
4. Accompanying hospital costs
5. 1-year mortality rate

Visiting a community neurologist was used as a parameter for continuity of care after hospital discharge and evidence-based secondary prevention. The utilization of this service within the first year after the index event was dichotomized. The frequency of recurrent events was used to assess the effectiveness of secondary prevention and measured by hospital diagnoses. All readmissions due to a stroke or TIA within the first year after the initial event were counted; secondary diagnoses of stroke indicating a complication during another procedure were excluded here. The number of all readmissions to a hospital in the first 12 months after initial stroke was included in the analysis, as well as the associated costs and average monthly hospital costs excluding costs of the initial treatment. Finally, the mortality rate only includes patients who had survived their initial stroke and were discharged after acute care.

### Statistical analysis

The comparability of the intervention and control groups, in addition to the matching criteria, was checked by analyzing the burden of comorbidities in both groups using the secondary diagnoses from the initial hospital documentation. The most relevant chronic diseases and comorbidities were analyzed both individually and in an aggregated index, counting the number of comorbidities following van den Bussche’s approach [28].

Outcome measures were descriptively analyzed and tested in bivariate and multivariable models accounting for the dependency of matched individuals in the control group. In the models, both arms of the intervention were analyzed as one group, since the potential effects of coordinated care in general was of interest. The potential effects of the intervention on the utilization of neurologist services was analyzed with mixed effects logistic regression models (random intercept models) with individual measurements of controls and treated patients as level-one units nested in matched pairs who were level two units. Effects on the occurrence of recurrent strokes were analyzed via mixed effects Poisson regression models (random intercept models) with individual measurements of intervention participants and controls (level-one units) nested in matched pair groups (level two units). For analyzing the frequency of hospital readmissions, we used negative binomial mixed regression models. Intervention effects on time to mortality were modeled with Cox regression models using R. To account for the within-cluster homogeneity of the clustered matched pairs a random effect term was incorporated. Hospital costs were analyzed in linear mixed models (random intercept models) with log-transformed costs as the dependent variable due to its skewed distribution as level-one units nested in matched pairs as level-two units. All models were adjusted for age, sex, pre-stroke long-term care dependency (yes/no), stroke type, and the number of comorbidities, since these factors are among the most important determinants of long-term stroke outcomes [29, 30]. Statistical assumptions of models were checked and confirmed. Hospital costs were analyzed in linear mixed models (random intercept models) with logarithmized costs as a dependent variable due to its skewed distribution as level-one units nested in matched pairs as level-two units. All event models (Poisson models, Cox models) accounted for the cases’ length of observation. A two-sided significance level of alpha = 0.05 was used. No adjustment for multiple testing was considered.

Analyses were done using SPSS 23, Stata 14, and R 3.

## Results

### Sample characteristics

**Fig. 1:**
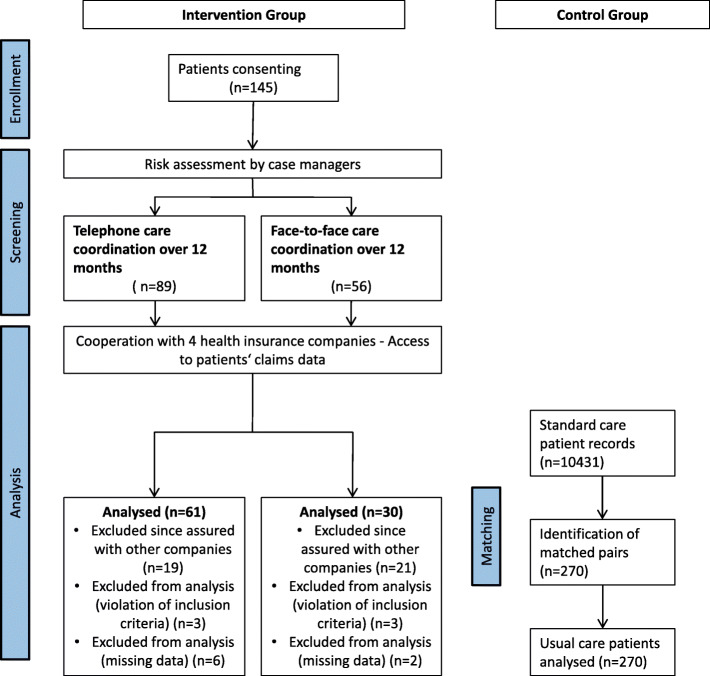
Patient flow of analyzed subjects

A total of *N* = 145 patients consented to participate in the program. There were sufficient claims data for 91 patients that could be used for the efficacy analysis (see flow chart, Fig. 1). Of the 91 stroke survivors, 60 received case management via telephone and 31 received face-to-face care coordination. As shown in Tables 1, 47.3% of the participants were female and the mean age was 75.3 years (SD: 9.2). There were no significant differences between the two intervention groups regarding age, sex, degree of care dependency before the stroke, or stroke type. However, in the face-to-face group, 13% more patients had a prior care dependency. When looking at the total length of stay in the acute-care hospital after the initial stroke, face-to-face participants stayed twice as long in the hospital compared to telephone participants.

**Table 1:** Description of study population

Characteristics	Intervention group (coordinated care)				Control group (usual care)	
	Telephone group ***n*** = 61	Face-to-face group ***n*** = 30	Standardized mean difference between intervention sub-groups	Total ***n*** = 91	Total ***n*** = 270	Standardized mean difference between intervention and control group
Sex, % (n)						
Female	42.6 (26)	56.7 (17)	0.28	47.3	47.4 (128)	< 0.01
Male	57.4 (35)	43.3 (13)		52.7	52.6 (142)	
Age, mean (SD)						
All	75.4 (9.3)	75.0 (9.2)	0.04	75.3 (9.2)	75.2 (9.5)	< 0.01
Female	78.0 (9.0)	77.1 (9.0)		77.6 (8.9)	77.6 (8.8)	
Male	73.5 (9.2)	72.3 (9.0)		73.2 (9.1)	73.0 (9.5)	
Dependency before strokein degree of severity, % (n)						
None	77.0 (47)	63.3 (19)	0.22	72.5 (66)	73.7 (199)	0.04
Degree 1	13.1 (8)	23.3 (7)		16.5 (15)	16.7 (45)	
Degree 2	8.2 (5)	13.3 (4)		9.9 (9)	8.5 (23)	
Degree 3	1.6 (1)	0.0 (0)		1.1 (1)	1.1 (3)	
Stroke type, % (n)						
Ischemia	82.0 (50)	83.3 (25)	0.40	82.4 (75)	81.9 (221)	0.04
Hemorrhage	8.2 (5)	13.3 (4)		9.9 (9)	9.6 (26)	
TIA	6.6 (4)	0,0 (0)		4.4 (4)	5.2 (14)	
Non-specified stroke	3.3 (2)	3.3 (1)		3.3 (3)	3.3 (9)	
Length of stay (days) in acute hospital (including direct transfers between hospitals/ wards), median (IQR)	10.0 (6.0–16.5)	20.5 (11.8–62.5)	0.63	12.0 (8.0–29.0)	13.0 (7.0–27.0)	0.20
Length of observation in months (max. 12 months), median (IQR)	12.0 (9.7–12.0)	12.0 (12.0–12.0)	0.16	12.0 (10.2–12.0)	12.0 (12.0–12.0)	0.80
Comorbidities, % (n)						
Diabetes	29.5 (18)	40.0 (12)	0.22	33.0 (30)	33.7 (91)	0.02
Hypertension	80.3 (49)	76.7 (23)	0.09	**79.1 (72)**	**66.7 (180)**	0.28
Ischemic heart disease	18.0 (11)	13.3 (4)	0.13	16.5 (15)	14.1 (38)	0.07
Renal insufficiency	19.7 (12)	16.7 (5)	0.08	18.7 (17)	13.7 (37)	0.14
Dementia	8.2 (5)	3.3 (1)	0.21	6.6 (6)	8.9 (24)	0.09
Atrial fibrillation	36.1 (22)	46.7 (14)	0.21	**39.6 (36)**	**25.9 (70)**	0.29
Cardiac insufficiency	6.6 (4)	6.7 (2)	0.01	6.6 (6)	11.1 (30)	0.16
Obesity	4.9 (3)	6.7 (2)	0.07	5.5 (5)	5.9 (16)	0.02
Asthma/COPD	4.9 (3)	0 (0)	0.32	3.3 (3)	5.9 (16)	0.13
Lipid metabolism disorders	44.3 (27)	40.0 (12)	0.09	**42.9 (39)**	**21.9 (59)**	0.46
Number of comorbidities, median (IQR)	3.0 (2.0–3.0)	3.0 (2.0–3.0)	0.02	3.0 (2.0–3.0)	2.0 (1.0–3.0)	0.28

Three matching cases from the standard care population could be identified for all but three participants. For these three participants, we could identify only two controls that matched in all six criteria instead of three. Here, we included these two controls in the analyses. Using frailty regression models and accounting for the matching by a random term, it is possible to use matched groups of patients with different numbers of matched controls. There was a total of *N* = 270 control cases. The intervention and control groups included censored cases with an observational time of fewer than 12 months, due to either death or missing data. Median follow up time for the intervention group was 12.0 months (IQR: 10.2–12.0) and 12.0 months (IQR: 12.0–12.0) for the control group (Table 1).

Ten common comorbidities were assessed, and the number of comorbidities per patient was calculated (Table 1). The intervention group had a median number of 3.0 diagnosed comorbidities (IQR: 2.0–3.0), the control group 2.0 (IQR: 1.0–3.0). The most common diagnosis in both groups was hypertension (IG: 79.1%; CG: 66.7%), followed by lipid metabolism disorders (IG 42.9%; CG: 21.9%), atrial fibrillation (IG: 39.6%; CG: 25.9), and diabetes (IG: 33.0%; CG: 33.7%).

### Outcome measures

In the first year post-stroke, the incidence rate per 100 person-months of visiting a community neurologist was 6.41 (95% CI: 5.00–8.20) in the intervention group, compared to 3.32 (95% CI: 2.73–4.04) in the control group (Table 2). The mixed effects logistic regression model revealed an odds ratio for the intervention group of 4.02 (95% CI: 2.35–6.86) in the unadjusted model, which means that patients in the intervention groups utilized a specialist more often than patients in the control group. In the adjusted multiple model, the odds ratio was even higher (OR = 4.75; 95% CI: 2.71–8.31). Younger age and lower number of comorbidities were additionally associated with higher probability of visiting a community neurologist (OR for age = 0.97; 95% CI: 0.94–0.99), OR for comorbidities = 0.77; 95% CI: 0.66–0.90) (Table 3).

**Table 2:** Outcomes – descriptive results

Outcome parameter	Intervention group, *n* = 91 Median follow up time (IQR): 12.0 (10.2–12.0)	Control group, *n* = 270 Median follow up time (IQR): 12.0 (12.0–12.0)
Utilization of outpatient specialist services, IR (95% CI) per 100 person months	6.41 (5.00–8.20)	3.32 (2.73–4.04)
Recurrent strokes within 12 months, IR (95% CI) per 100 person months	0.79 (0.21–2.92)	1.22 (0.77–1.93)
Readmissions within 12 months, IR (95% CI) per 100 person months	4.03 (2.58–6.28)	9.56 (7.99–11.43)
Costs of readmissions per 6 months in €, median (IQR)	0 (0–1910)	938 (0–4375)
Survival (within 12 months), KME (95% CI)	0.92 (0.87–0.98)	0.90 (0.87–0.94)

**Table 3:** Outcomes – Unadjusted separate and adjusted multiple models

Utilization of outpatient specialist services: Mixed effects logistic regression (*n* = 361 individuals / 91 matched groups)				
	Unadjusted separate models		Adjusted multiple model	
	**Odds ratio** (95% CI)	***p*-value**	**Odds ratio** (95% CI)	***p*-value**
Intervention (ref.: controls)	**4.02 (**2.35–6.86)	< 0.001	**4.75** (2.71–8.31)	< 0.001
Age (years)	**0.97** (0.95–0.99)	0.004	**0.97** (0.94–0.996)	0.020
Female Sex (ref.: males)	0.76 (0.50–1.15)	0.191	0.87 (0.54–1.39)	0.560
Pre-stroke Care Dependency	**0.58** (0.36–0.94)	0.026	0.78 (0.39–1.37)	0.389
Stroke Type				
- Ischemia (Ref.)	1		1	
- Hemorrhage	1.32 (0.65–2.68)	0.446	1.33 (0.67–1.01)	0.464
- TIA	0.79 (0.29–2.11)	0.638	0.67 (0.23–1.96)	0.469
- non-specified	1.24 (0.39–4.00)	0.715	1.01 (0.29–3.60)	0.983
Number of comorbidities	**0.81** (0.71–0.92)	0.002	**0.77 (**0.66–0.90)	0.001
Recurrent strokes: Mixed effects Poisson regression (*n* = 361 individuals / 91 matched groups)				
	Unadjusted separate models		Adjusted multiple model	
	**IRR** (95% CI)	***p*-value**	**Odds ratio** (95% CI)	***p*-value**
Intervention (ref.: controls)	0.78 (0.39–1.57)	0.491	0.76 (0.38–1.54)	0.451
Age (years)	1.01 (0.97–1.04)	0.757	1.02 (0.98–1.05)	0.301
Female Sex (ref.: males)	**0.51** (0.27–0.94)	0.031	**0.49** (0.26–0.93)	0.028
Pre-stroke Care Dependency	0.70 (0.33–1.48)	0.349	0.67 (0.31–1.44)	0.309
Stroke Type				
- Ischemia (Ref.)	1		1	
- Hemorrhage	1.32 (0.54–3.20)	0.541	1.23 (0.52–2.90)	0.636
- TIA	0.39 (0.05–2.92)	0.362	0.36 (0.05–2.6)	0.308
- non-specified	1.82 (0.53–6.16)	0.339	1.31 (0.40–4.34)	0.664
Number of comorbidities	1.00 (0.84–1.19)	0.973	1.03 (0.86–1.23)	0.743
Hospital readmissions: Mixed effects negative binomial regression (*n* = 361 individuals / 91 matched groups)				
	Unadjusted separate models		Adjusted multiple model	
	**IRR** (95% CI)	***p*-value**	**IRR** (95% CI)	***p*-value**
Intervention (ref.: controls)	**0.45** (0.31–0.65)	< 0.001	**0.42** (0.29–0.61)	< 0.001
Age (years)	1.01 (1.00–1.03)	0.109		0.181
Female Sex (ref.: males)	1.01 (0.75–1.36)	0.940	0.89 (0.66–1.20)	0.452
Pre-stroke Care Dependency	**1.62** (1.17–2.25)	0.004	1.38 (0.98–1.94)	0.062
Stroke Type				
- Ischemia (Ref.)	1		1	
- Hemorrhage	1.03 (0.62–1.69)	0.916	1.10 (0.68–1.79)	0.703
- TIA	0.96 (0.49–1.85)	0.899	1.00 (0.53–1.89)	1.000
- non-specified	0.67 (0.28–1.64)	0.382	0.69 (0.29–1.66)	0.407
Number of comorbidities	**1.12** (1.03–1.23)	0.010	**1.12** (1.03–1.22)	0.012
Mortality: Frailty survival model (*n* = 361 individuals / 91 matched groups, 38 events)				
	Unadjusted separate models		Adjusted multiple model	
	**Hazard ratio** (95% CI)	***p*-value**	**Hazard ratio** (95% CI)	***p*-value**
Intervention (ref.: controls)	0.74 (0.32–1.68)	0.57	0.65 (0.28–1.49	0.386
Age (years)	**1.11** (1.06–1.16)	< 0.001	**1.11** (1.05–1.16)	< 0.001
Female Sex (ref.: males)	0.99 (0.46–2.10)	0.970	0.56 (0.26–1.23)	0.150
Pre-stroke Care Dependency	**2.93** (1.38–6.24)	0.005	1.54 (0.68–3.506)	0.300
Stroke Type				
- Ischemia (Ref.)	1		1	
- Hemorrhage	1.32 (0.42–4.13)	0.630	1.37 (0.46–4.11)	0.570
- TIA	/	/	/	/
- non-specified	0.76 (0.08–7.39)	0.810	1.23 (0.97–11.60)	0.860
Number of comorbidities	**1.25** (1.02–1.52)	0.028	1.19 (0.97–1.46)	0.093
Hospital readmission costs: Linear mixed model (*n* = 361 individuals / 91 matched groups)				
	Unadjusted separate models		Adjusted multiple model	
	**β** (95 % CI)	***p*-value**	**β** (95 % CI)	***p*-value**
constant	**–**		**5.50** (4.66–6.34)	< 0.001
Intervention (ref.: controls)	**−0.34** (−0.54 - -0.14)	0.001	**−0.37** (−0.57 - -0.18)	< 0.001
Age (years)	**0.01** (0.001–0.02)	0.024	**0.01** (− 0.002–0.02)	0.131
Female Sex (ref.: males)	−0.06 (− 0.24–0.11)	0.480	−0.12 (− 0.30–0.05)	0.158
Pre-stroke Care Dependency	**–**		**0.28** (0.07–0.49)	0.009
Stroke Type				
- Ischemia (Ref.)				
- Hemorrhage	0.20 (−0.10–0.51)	0.185	0.24 (− 0.06–0.53)	0.115
- TIA	− 0.05 (− 0.46–0.36)	0.802	0.03 (− 0.37–0.43)	0.885
- non-specified	−0.01 (− 0.50–0.50)	0.980	0.04 (− 0.45–0.53)	0.874
Number of comorbidities	**0.07** (0.02–0.12)	0.012	**0.06** (0.01–0.12)	0.005
Models with log-transformed costs as dependent variable				

In the intervention group, 9.9% suffered from another stroke within 12 months compared to 13% in the control group (values not shown in tables). As indicated by the bivariate analysis (Table 2), the mixed effects Poisson regression showed no significant effect of the intervention (Table 3). The multiple model showed that women had a recurrent stroke less frequently than men (IRR = 0.49; 95% CI: 0.26–0.93) (Table 3).

Readmissions to a hospital were recorded for 37.4% in the intervention group and 57.4% in the control group (values not shown in tables). Participants had an incidence rate of 4.03 (95% CI: 2.58–6.28) readmissions per 100 person-months, standard care patients 9.56 (95% CI: 7.99–11.43) (Table 2). In the mixed effects Poisson regression, the intervention group had significantly fewer readmissions (IRR = 0.46; 95% CI: 0.34–0.62). In the multiple model, the effect of the intervention on readmissions was similar to the effect in the unadjusted model. Patients with a higher number of comorbidities were admitted to ahospital more frequently (Table 3).

The number of readmissions was accompanied by substantially lower hospital costs in the intervention group (median 6 months costs: €0, IQR: €0–1910) compared to those in the control group (median 6 months costs: €938, IQR: €0–4375). This difference was also confirmed in the linear mixed model (Table 3).

The 12-month survival rate for the intervention group was 0.92 (95% CI: 0.87–0.98) compared to 0.90 (95% CI: 0.87–0.94) in the control group (Table 2). The frailty survival model showed no significant effect of the intervention (Table 3).

## Discussion

Our study investigated the potential effects of a coordinated care program for community-based stroke survivors within 12 months after their initial stroke. Whereas the intervention was associated with an increased rate of patients visiting a community neurologist, the number of recurrent strokes did not decrease. Nevertheless, hospital readmissions in general were significantly less common in the intervention group than the control group receiving standard care. Higher numbers of hospital readmissions were accompanied by higher hospital costs in the control group. Differences in mortality were not observed.

As patients were partially recruited by participating community neurologists (16% of the participants), the effect of the intervention on the outpatient specialist utilization rate might be overstated. However, this explains only up to half of the 32% difference between the intervention and control group.

Study group characteristics indicate that participants were comparable with German stroke patients regarding sex, age, and stroke type distribution [31, 32]. Only TIA patients were considerably underrepresented compared to stroke population distributions [31, 32], which may be explained by their lower burden of functional impairments and their lower demand for support in the recovery process. TIA patients were defined as part of the target population due to their increased care needs and the high risk of incident recurrence.

Comparisons of our results with previous studies may only be drawn carefully. There are numerous factors – including details of care concepts, structural conditions, regional characteristics, study design, and outcome measures – that vary considerably between studies with similar research foci.

In general, post-stroke intervention studies have mostly focused on patient-centered outcomes such as motor functions or quality of life. In a Cochrane review on the efficacy of health care workers supporting stroke survivors after discharge from the hospital, Ellis et al. found mixed evidence for the effectiveness of these outcomes. Even though they were interested in assessing resource use, this was not possible due to a lack of comparable data [33].

Apart from the problem of comparability, discussing our results in the context of previous research elucidates the problem of replicability [34], which is especially valid for complex community interventions.

### Secondary Prevention & Rate of recurrent events

While secondary stroke prevention has become a central aim of post-stroke care, only a few comparable studies have reported outpatient neurologist visits. Two care coordination interventions in Canada and Germany found no differences between intervention and control groups [35, 36]. However, in both approaches, enabling access to health services was not the primary aim. In Canada, there was no need to increase utilization as the rate was already at 82% [35]. Most studies with a focus on secondary prevention used clinical measures as outcomes. A Cochrane review found moderate-quality evidence that organizational interventions improved some modifiable risk factors, e.g., blood pressure and lipid profiles [37]. Yet, no evidence of a reduction in the incidence of recurrent events associated with the intervention was found.

A stroke-treatment network, including three specially trained nurses focusing on secondary prevention in Dijon, France, found a significantly lower rate of recurrent events when comparing the numbers 8 years before and 5 years after establishing the network [38]. Even though study design and methodology are very different from this and most other studies’ approaches, the Dijon study has the advantage of a much longer observation time. The effect of complex interventions might become measurable only after a couple of years. The increased risk of recurrent events persists for at least 10 years after a first stroke [39].

Our hypothesis that increasing patients’ utilization of outpatient specialist services and coordination of services by case managers would lower the recurrence rate within 1 year was not confirmed. In the German health care system, general practitioners are responsible for follow-up care in general. However, community neurologists are specialized in secondary prevention, and family doctors are supposed to cooperate with neurologists in caring for their patients [40]. Yet, the utilization of neurologists is low despite their crucial role in post-stroke care. This might be due to information deficits, patients’ low risk awareness [41], and the associated high organizational and logistic burdens. Community neurologists often have long waiting lists or do not admit new patients at all. Moreover, they are distributed unevenly across regions and city areas, so patients in certain neighborhoods must overcome long distances to seek care. For many stroke patients, these burdens seem to exceed their personal threshold. Enabling access to community neurologists therefore seemed to be the first crucial step to improve secondary prevention and to prevent recurrent strokes.

### Readmissions

While no effect on the rate of recurrent strokes was observed, there was a significantly lower rate of hospital readmissions for any reason in our intervention group. Besides the risk of recurrent events, stroke survivors are at a high risk of being readmitted to a hospital [42]. Falls in stroke patients, for instance, are very common, and often lead to emergency department or hospital treatments [43], which could often be prevented [44]. Decreased hospitalization rates were also observed in some comparable intervention studies. In the aforementioned Canadian study [35], case management was conducted in a similar way as in our study. While referring to a shorter period of time, the authors also found an effect on readmissions, albeit theirs was smaller than the effect we observed. The number of both unplanned and scheduled readmissions was slightly lower in the intervention group at 6 months in the Canadian study [35].

In a German study by Saal et al., there was no significant difference in the (patient-reported) readmission rate between the intervention and control groups [36]. This might be due to their focus on physical functioning rather than on overall care coordination.

In Denmark, additional stroke aftercare by either physicians or physiotherapists had significant effects on readmission rates at 6 months [45]. Despite the difference in the professions supporting the patients (physicians and physiotherapists vs. nurse case managers), the effects on readmissions rates are similar in size compared to ours.

While the problem of high readmission rates concerns not only stroke survivors but also patients with chronic and complex diseases in general, evidence on the potential of care interventions remains incoherent [46]. An umbrella review covering a large body of studies on integrated care intervention concluded that the mechanisms of successful interventions to prevent readmissions are still not sufficiently understood [46].

With regard to our project, it might be assumed that coordinated community care could prevent many unnecessary and unplanned hospitalizations. The program seemed to improve the management of general risk factors and general exacerbations of health status, rather than specific stroke-related factors.

### Costs

Only a few post-stroke intervention studies have taken costs as an outcome into account, finding both no cost differences [47] and cost-effectiveness [48, 49]. The positive economic effects of a support program in Quebec were primarily due to fewer readmissions [49]. The authors assumed that through their home care visits, patients’ health problems could be identified and dealt with before they exacerbated and had to be taken to the hospital – an interpretation that also seems reasonable for our study.

### Mortality

Most intervention studies considering mortality as an outcome did not see an effect of their interventions, neither at three [50], six [45], nor 12 months [50, 51]. Ertel et al. observed a positive effect of their intervention on mortality at an average of 47 months post-stroke. This could indicate a methodological problem: the fundamental effects of complex interventions, such as reduced mortality, might be seen only after a long-term observation exceeding the typical 12-month follow-up.

### Strengths and limitations

When interpreting the results of our study, several strengths and limitations must be considered. One limitation of our study is the relatively small and specific setting, namely that it took place within one district in Berlin. The intervention was implemented by a health care network, that had evolved over more than a decade. Our results thus cannot be simply extrapolated to different settings such as rural areas and non-German healthcare systems, or to the general stroke population. Due to ethical and practical considerations, we used matched pairs from a large cohort to compile a quasi-control group instead of using a randomized control group.

Using claims data involves methodological advantages as well as disadvantages. Such data does not include patient-reported outcomes such as quality of life or satisfaction with health services. Moreover, they lack clinical data beyond ICD-10 logic or information on the individual’s psychosocial background, for instance, the socioeconomic status, social support, activities of daily living and laboratory data. For some characteristics, claims data only provide crude surrogates such as the length of stay as a parameter for the severity of stroke. We cannot rule out a difference in the severity of stroke between the intervention and control groups. However, there is also no indication that there was a bias in this regard. The matching approach and the scope of the recorded patient information only allow for a limited set of covariate variables to be involved, in order to control the risk of selection bias. The comparative analyses of comorbidities yielded a few deviations in the control group. However, the comparison between the intervention and control group regarding comorbidities showed a higher prevalence in the intervention group only. The remarkable difference in the number of readmissions thus cannot be attributed to the difference in the burden of morbidity.

The study focused on specialist utilization and hospitalizations as outcomes. However, depending on guidelines and patients’ needs, further relevant disciplines such as nursing care and physical or speech therapy also need to be involved in care planning. As described above, our program encompassed all of these aspects of post-stroke stroke care.

One of the strengths of evaluations based on claims data is that they provide reliable and exhaustive records of whether, when, and at what cost patients received treatments covered by their health insurance. They are robust against dropouts, are free of any recall bias, and are not bound to single recruitment centers.

Another strength of our data lies in the variety resulting from our use of data from four different health insurance companies, which reduces the risk of selection bias related to the different socioeconomic profiles of company clientele.

## Conclusion

Evidence pointing to the effectiveness of complex community- based interventions for stroke survivors is mixed. Some studies show relevant benefits in at least some areas, while other studies show little or none.

Our findings indicate that care coordination is a potentially effective instrument to support stroke survivors as they organize and utilize the necessary and available health services in the community in a coherent way.

Care coordination helps patients to access appropriate health services and may decrease the use of inadequate and expensive inpatient care. At the same time, bottom-up networking of health care providers in the interest of patient-oriented health care might be a reasonable starting point to implement care coordination. However, more tailored concepts are needed to address stroke patients’ specific needs and risks. Further large-scale studies are required to verify our findings. Finally, future studies will also benefit from complex study designs that are able to identify the most effective elements of complex interventions and help to understand how their mechanisms work.

## Data Availability

The data that support the findings of this study are available from the four cooperating health insurance companies (see acknowledgments); however, restrictions apply to the availability of these data, which were used under a license for the current study, and so are not publicly available. The data are available from the corresponding author upon reasonable request and with the permission of the health insurance companies.

## Abbreviations

CG: Control group
CI: Confidence interval
IG: Intervention group
IQR: Interquartile range
IR: Incidence ratio
IRR: Incidence rate ratio
KME: Kaplan-Meier estimator
OR: Odds ratio
TIA: Transient ischemic attack
